# Zinc Recovery from a Water Supply by Reverse Osmosis Operated at Low Pressures: Looking for Sustainability in Water Treatment Advanced Processes

**DOI:** 10.3390/membranes14060131

**Published:** 2024-06-05

**Authors:** Paola Andrea Alvizuri-Tintaya, Paul d’Abzac, Vanesa G. Lo-Iacono-Ferreira, Juan Ignacio Torregrosa-López, Jaime Lora-García

**Affiliations:** 1 Centro de Investigación en Agua, Energía y Sostenibilidad, Universidad Católica Boliviana San Pablo, La Paz, Bolivia; 2 Centro de Investigación en Ciencias Exactas e Ingenierías, Universidad Católica Boliviana San Pablo, Cochabamba, Bolivia; pdabzac@ucb.edu.bo; 3 Project Management, Innovation and Sustainability Research Center (PRINS), Universitat Politècnica de València, Alcoy Campus, Plaza Ferrándiz y Carbonell, s/n, 03690 Alcoy, Spain; valoia@upv.es; 4 Research Institute for Industrial, Radiophysical and Environmental Safety (ISIRYM), Universitat Politècni-ca de València, Plaza Ferrándiz y Carbonell, s/n, 03690 Alcoy, Spain; jitorreg@iqn.upv.es (J.I.T.-L.); jlora@iqn.upv.es (J.L.-G.)

**Keywords:** wastewater, reverse osmosis, added value, metal recovery

## Abstract

Achieving sustainability in life involves increasing efforts to recover resources. This research proposes the recovery of Zn from the Milluni lagoons, an important water supply for Bolivia, where high concentrations of Zn have been identified that exceed permitted limits, exposing a risk to health and ecosystems. The application of reverse osmosis (RO), operated with low pressures, is proposed as a first stage for the concentration of Zn and subsequent recovery of this metal through chemical precipitation. The aim was to maintain the separation efficiency of the RO operated at low pressures without presenting operational problems. As a result, 98.83% metal concentration was achieved with a laboratory-scale pilot system. The above means an important potential for large-scale Zn concentration, apart from orienting the RO toward sustainability by working with low pressures that reduce energy costs during its operation. This study can be used as a valuable reference for the advancement of sustainable technologies in the field of water treatment that simultaneously allow the recovery of resources to promote a circular economy. Finally, this study exposes an alternative for regions with heavy metal water contamination in Bolivia and worldwide.

## 1. Introduction

Water is essential for life and development on Earth. However, the unequal distribution of water worldwide, increased population requiring more water for consumption, and climate change have resulted in a global water crisis. Furthermore, during the last decade, growing industrialization has put pressure on water resources, thus reducing the availability of water for consumption [1]. Drinking water is indisputably linked to public health and this, in turn, to sustainable development [2]. This is why caring for water has become a priority in Goal 6, “Clean Water and Sanitation”, within the Sustainable Development Goals (SDGs) that frame the 2030 agenda [3].

Considerable amounts of time, effort, and money are invested in controlling, protecting, managing, and restoring surface water resources, recognizing the importance of water for the social well-being and economic development of nations [4,5,6]. Environmental standards and regulations are becoming more stringent, requiring more sophisticated and effective treatments to ensure safe liquid discharges [7]. Treatment plants have a series of processes to reduce contaminants in the water, but waste that can be reused is also generated during treatment. However, the different water treatment plants are not yet seen as mechanisms for using byproducts generated during treatment [8]. Target 12.2 of Goal 12, “Responsible consumption and production”, of the SDGs, indicates that efficient management of natural resources can be achieved through the recovery of resources that can be reintroduced into the production cycle [3]. The above can be achieved with the application of a circular economy model.

The circular economy focuses on improving sustainability by restructuring consumption and production patterns [9]. The circular model seeks to ensure that waste is used through its reinsertion into the production cycle or commercialization, generating added value to the recovered waste [10]. The above causes a positive impact on the production chain and reduces the overexploitation of raw materials. The Ellen McArthur Foundation has proposed some of the basic principles of the circular economy, which have been introduced in different international regulations, for example, in the EU Directive 2008/98/EC on Waste [11,12]. The EU Directive 2008/98/EC recommends the “waste hierarchy” for waste management, with an order of priority from the most preferred option of “prevention” at the top to the least preferred option, “disposal”, at the bottom [12]. However, there are limitations in the “waste hierarchy” as a facilitator of sustainable development. For this reason, the “hierarchy of resource use” has been proposed, which could reduce the consumption of virgin natural resources and the consequent concomitant environmental degradation with an increase in the efficiency of global resources [13]. The “hierarchy of resource use” has three main parts: replacement, reduction, and recovery; the last part is where the opportunity for Wastewater Treatment Plants (WWTPs) to be transformed into Water and Resource Recovery Facilities (FRRs) is located. The above will be achieved through the implementation of a circular economy model.

A combination of more sustainable thinking, an increase in the cost of solid waste disposal, and the decrease in available natural resources has led to a growing interest in the transition from a linear to a circular economy in different production facilities, as well as in WWTPs [14,15,16]. With a circular economy, WWTPs can be transformed into W&RRFs and recover resources such as water, energy, metals, biosolids, and nutrients [17]. In 2021, UN-Habitat and the WHO presented the progress on water treatment worldwide, listing the countries that had reported information on treating their wastewater, including safely treated wastewater, that is, which receive at least one secondary treatment [18]. In this sense, WWTPs’ existence was shown, and this is where the waste recovery concept could be introduced. The growing public awareness about the scarcity of resources and the added value of recovered waste will be the incentive for the transformation of WWTPs [19]. However, it must be taken into account that the configuration of current processes in WWTPs has been aimed at reducing water contaminants and not for waste recovery, which will be the first challenge for transforming WWTPs into W&RRFs [20].

### Zinc, Removal, and Recovery

The atomic number of zinc is 30, and its symbol is Zn; this metal is found in group IIb on the periodic table of elements, along with two toxic metals, cadmium and mercury. The Maximum Contaminant Level (MCL) for drinking water for Cd is 0.005 mg/L and Hg 0.002 mg/L [21]. However, Zn is considered relatively non-toxic for humans [22]. A health-based guideline value is not required for Zn, as it is an essential trace element for humans and all organisms [23]. However, drinking water quality deteriorates at Zn concentrations higher than 4 mg/L, and an undesirable astringent taste is detected [24]. With extremely high Zn intake, manifestations of toxicity symptoms such as nausea, vomiting, epigastric pain, lethargy, and fatigue occur [25]. For this reason, the Environmental Protection Agency (EPA) of the United States has established a maximum concentration of 5 mg/L as a limit value for Zn in drinking water [21]. The Bolivian Standard 512 for drinking water is consistent with the Zn concentration value established by the EPA [26].

**Figure 1:**
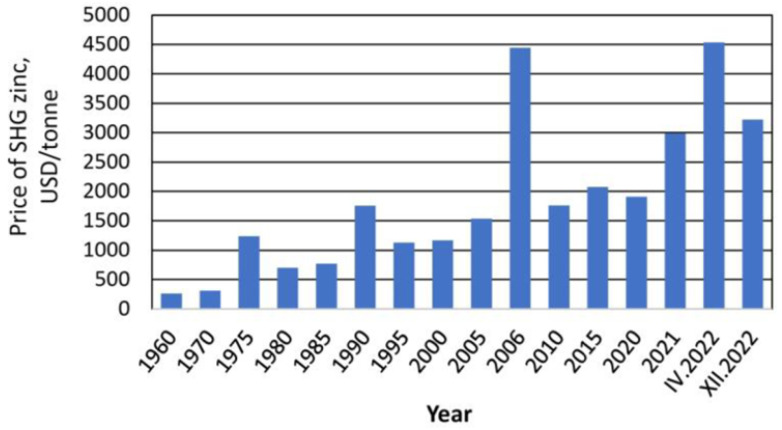
Average prices for zinc worldwide from 1960 to 2022. Source: [27].

Currently, Zn is one of the most important metals for the development of technology [27]. World Zn reserves are estimated at 250 million tons and are projected only to last the next 17 years [28]. The increasing demand for Zn may limit its future availability, and the cost will be higher. Figure 1 presents the variation in the price per ton of Zn over time.

In 2021, the demand for Zn reached 14 million tons, but in the second part of 2022, its demand decreased due to the economic effects of COVID-19 [27]. By 2023, according to recently compiled data, the price trend continued to decrease, reaching a value of 2500 USD/ton [29]. According to the International Zinc Association, 25% of global Zn consumption comes from secondary or recycled sources [28]. The production of Zn from secondary or recycled sources brings measurable benefits, such as saving primary and fossil resources, reducing storage and losses of Zn, valorizing waste, and mitigating effects on the environment and health [27]. Raw material conservation is one of the most attractive global concerns of the 21st century [30]. For this reason, searching for new unconventional sources of Zn is an imminent current and future need.

Water treatment processes in WWTPs are beginning to be perceived as a traditional role of systems for removing contaminants and in a new role associated with recovering resources and energy [20]. However, not all water treatment processes have the same potential to recover metals [7]. Advanced treatments have been proposed to recover metals in W&RRFs [31]. Several treatments have been used to remove metals from water; among those presented, the best results are adsorption, biosorption, ion exchange, electrocoagulation, and membrane filtration, specifically reverse osmosis (RO) [32,33]. The adsorption, biosorption, and ion exchange processes are more expensive in capital because they require very large interfacial treatment areas; they also require regeneration of the materials that interact in the process, which is also expensive [31]. The RO presents a simple, modular system that does not require large quantities of chemicals to clean the membranes [34].

RO is operated with membranes with a pore size < 1 nm, which works on the principle of size exclusion and solution diffusion through a semipermeable membrane. The typical RO design is configured by modules with elements in series to achieve high water conversions, close to 85%. The RO works under a pressure gradient over the osmotic pressure of the solution to obtain a solvent flow sufficient to carry out this operation on an industrial scale [35]. A significant advantage of RO over other technologies is its ability to concentrate ionic contaminants into one of its outputs “concentrated” [34]. In this sense, RO has positioned itself compared to other advanced treatments due to its high selectivity for separating contaminants at the ionic level present in water [35]. However, the high energy costs during its operation have limited its applicability in many contexts [36]. Membrane fouling, caused by the accumulation of impurities, continues to be a challenge in technologies that use membranes. However, it has also been observed that working at low pressures reduces fouling [37]. Despite the limitations, membrane technologies in resource recovery are promising [38].

Some studies demonstrated a high efficiency of up to 94% in removing Zn from aqueous solutions by RO [39,40]. However, none of the above have considered optimizing RO operating parameters, such as the work pressure to reduce energy costs. Operating RO at low pressures is interesting as it can concentrate Zn, which could lead to metal recycling. This research aims to evaluate the removal of Zn from water by RO operated at low pressures to establish sustainability within this process operational parameter. In addition, Zn recovery alternatives will be studied as a second stage after the application of RO, considering the particularities of the case study in Milluni, Bolivia.

## 2. Materials and Methods

### 2.1. Study Area

Milluni is approximately 4600 m above sea level in the Bolivian tin belt [41]. This micro-basin has an area of 40 km^2^, is part of the Altiplano basin system, and presents extreme climatic conditions typical of the Altiplano area [42]. Due to its geomorphology, mining was the main activity developed in Milluni [43]. The most active period of mineral exploitation was recorded between 1940 and 1990 [44]. Although widespread mining activities ceased approximately 20 years ago, the impact of mining waste on water quality remains a serious national environmental problem [45,46]. Another problem affecting water quality in this area is small-scale and often illegal mining activities, about which precise information is unavailable [47].

**Figure 2:**
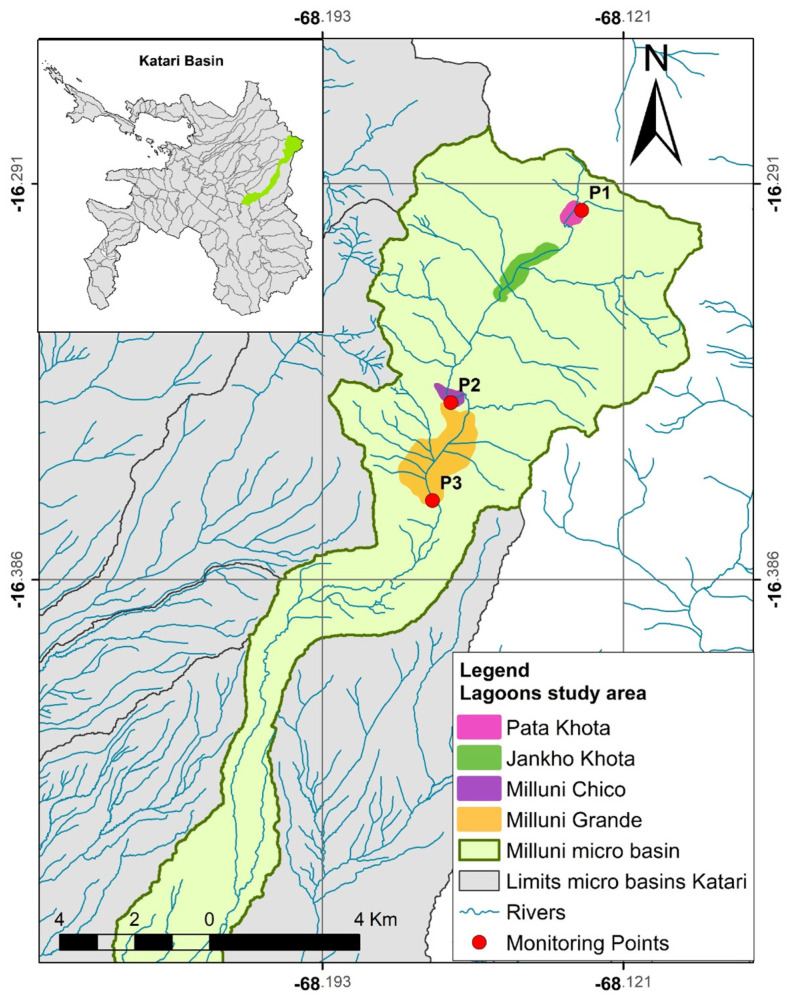
Lagoons in the upper part of the Milluni micro-basin. Source: Redrawn from [49].

In the upper part of the Milluni micro-basin are four lagoons: the Pata Khota Lagoon, Jankho Khota Lagoon, Milluni Chico Lagoon, and Milluni Grande Lagoon [48]. The first two are natural lagoons that receive water from the melting of the Huayna Potosí Mountain. The third lagoon, called Milluni Chico, is an artificial lagoon of irregular shape, which was built to capture the drainage waters of the mine to prevent them from entering and contaminating the fourth lagoon. However, this does not work at all. The fourth lagoon, Milluni Grande, is where the storage dam for all the water in the area is located; it has a capacity of 1,000,000 m^3^ and a surface of 2,450,000 m^2^ [45,48]. Figure 2 shows the upper part of the Milluni micro-basin and the location of the lagoons described above.

Water from the Milluni Grande Dam is used to meet the drinking water demand of approximately 500,000 inhabitants of La Paz and El Alto, two of the most populated cities in Bolivia [50]. Milluni water goes through pretreatment and is distributed to two water treatment plants of the Public Social Water and Sanitation Company (EPSAS) before entering the distribution network [51]. However, water treatment plants do not have specific treatments to eliminate metals; the plants have pretreatment, primary treatments (physical and chemical), and disinfection, but no advanced treatment [52]. In this sense, water quality for consumption is not guaranteed

### 2.2. Design of Water Quality Monitoring Program for Milluni

The Milluni micro-basin is part of the Katari macro-basin. The Katari macro-basin was defined as a strategic basin by the National Basin Plan of Bolivia [53]. The Katari Basin Management Unit is the body in charge of monitoring the water resources of the macro-basin from 2006 to the present. In this sense, there is historical data on the water quality of the upper part of Milluni, but a specific monitoring program has not been implemented. Through water quality monitoring in Milluni, the presence of certain metals, such as Zn, was identified in concentrations far outside the established limits [52]. The above may be interesting for metal recovery purposes.

A sampling protocol was formulated to obtain data on Zn concentrations in the study area, taking international standards as a basis. The UNEP/WHO monitoring program design guide [54], ISO 5667-1 Guidance on the design of sampling programs and sampling techniques [55], and ISO 5667-4 Guidance on lake sampling natural and man-made were considered [56]. The sampling results are presented in Section 3. The main components of the sampling protocol are detailed in Table 1.

The data obtained from Milluni monitoring will allow identifying the place where the Zn concentration is highest and, therefore, where a recovery strategy for this metal should be applied.

### 2.3. RO Pilot System

**Figure 3:**
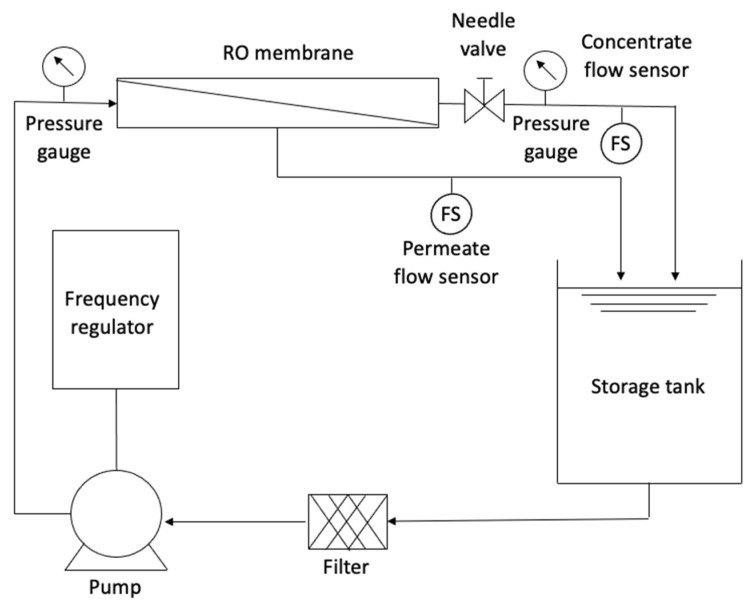
Technical scheme of the RO system.

The RO pilot system was mounted on an aluminum and stainless steel bracket. The system operated in a closed circuit, which means that the water from the storage tank was pumped to the RO module (membrane), and there were two outlets (concentrate and permeate) that were returned to the storage tank. Figure 3 presents a technical scheme of the RO system.

The main component of the pilot was a spiral-wound polyamide membrane with an active area of 25 m^2^, having a permeate rate of 2.84 m^3^/d with a concentrate rejection of up to 99.3%, according to manufacturer specifications. The maximum working pressure is 4.14 × 10^6^ Pa, and the minimum proportion of concentrate is 8%, according to the manufacturer. Since RO needs a driving force, a multistage centrifugal/electric pump with a power of 2 HP was used, with a maximum operating pressure of up to 5.00 × 10^5^ Pa. The pumping system was configured using a frequency regulator that allowed the manipulation of the inlet flow to the system. A water filter capable of retaining any suspended material was incorporated before the pump to protect the membrane module.

To evaluate the behavior of the membrane, the flows of the two outlets (permeate and concentrate) were permanently observed. For this purpose, flow sensors of 0.5 to 5 L/min for the permeate and another of 1 to 25 L/min for the concentrate were installed. The sensors were programmed on an Arduino board connected to a computer, displaying the data flow in real time. Pressure gauges located before and after the membrane module were used to control the system’s pressure loss and the working pressure. Additionally, a needle valve was used to control the flow and pressure in the system.

The permeate test is an initial test the manufacturer recommends to establish initial permeate flow conditions when the membrane is new/clean. This test lets a solution of 1500 mg/L of NaCl pass through the membrane at a pressure of 1034.21 KPa; the permeate flow data must be taken after 30 min of operation. The permeate data evaluate the decrease in membrane permeability over time, usually due mainly to fouling. Initial permeate data also help monitor the effectiveness of cleaning sessions since, after each cleaning session, the permeate should return to its original values. The membrane was cleaned with citric acid from BIOPACK^®^ at the end of each experimental test to avoid altering the behavior of the membrane with any type of fouling. The components of the system are detailed in Table 2.

### 2.4. Operational Conditions for the RO Pilot System

The operating variables that were taken were pressure “P”, income flow “Q_f_”, and the concentration of synthetic water “C_f_”. For each variable, three levels of operation were considered: low, medium, and high. The response variables we sought to analyze were the average analytical flux “J_v_” and the rejection index “R_o_”. To determine the number of tests necessary for the operating variables to interact with each other with their three levels, a design of experiments was carried out with the Statgraphics Centurion XVIII program using the “Design of Experiments Assistant” application. The type of experimental design used was a factorial fraction with three levels. The number of representative trials for the experiment was nine. For each test, the levels of the operating variables were different, allowing the process to be evaluated in different scenarios.

The operating levels for the variable P were below the manufacturer’s range; the work was realized with 5.00 × 10^5^ Pa (low level), 7.50 × 10^5^ Pa (medium level), and 1.00 × 10^6^ Pa (high level). The above is for operating in sustainable energy conditions and trying not to reduce separation efficiency. For the income flow “Qf”, there were also three levels: 0.8 × 10^−4^ m^3^/s (low level), 1.1 × 10^−4^ m^3^/s (medium level), and 1.4 × 10^−4^ m^3^/s (high level).

Regarding Co, the operation was performed within the ranges stipulated by the manufacturer but established three levels of operation to find the better one. The Co levels were selected according to the Zn concentrations and conductivity conditions found in previous monitoring of natural waters in the study areas. For the experimental part, synthetic water was used, prepared by adding a solution of Zn in distilled water combined with sodium chloride (NaCl); these are presented in Table 3. NaCl was used to replicate the conductivity conditions of the study water.

The Zn used was in 1000 μg/mL solutions from Inorganic Ventures^®,^ Virginia VA, EEUU. The NaCl used was from BIOPACK^®^ with a purity > 99%. It should be noted that the high concentration level exceeds four times what is stipulated for the Zn content in international and national standards [25,26].

### 2.5. Performance of RO Pilot System

A mathematical and statistical analysis of the process was carried out to evaluate the performance of the RO operated at low pressures. The mathematical part was carried out to understand the behavior of the membrane under pressure conditions outside the range established by the supplier, seeking to maintain the efficiency of Zn removal operating with low pressures. The statistical part was performed to optimize the operating conditions and thus find the best scenario for the recovery of Zn in a second stage after the application of RO. The following sections describe how each part was developed.

#### 2.5.1. Mathematical Evaluation of the RO Process

First, some concepts and relevant variables in the RO process are presented. The principle of conservation of mass is presented in Equations (1) and (2); these equations help to analyze the balance that must exist between the input and the two outputs of the process.

Q_f_ × C_f_ = Q_c_ × C_c_ + Q_p_ × C_p_ (1)

Q_f_ = Q_b_ + Q_p_ (2)

where

Q_f_ = feed flow,
C_f_ = feed concentration,
Q_c_ = concentrate flow,
C_c_ = concentration of the concentrate flow,
Q_p_ = permeate flow, and
C_b_ = concentration of the permeate flow.

The relationship between Q_p_ and J_v_ is shown in Equation (3). J_v_ represents the flux of the solvent, and it is an important variable that indicates the behavior of the membrane during the separation process, which is driven by a pressure differential.

J_v_ = Q_p_/S (3)

where

S = effective area of the membrane.

The recovery “y”, representing the water production capacity, is the fraction of the feed flow that passes through the membrane, also called the permeate flow. The greater this flow, the greater the production capacity of the RO system. This value is determined with Equation (4).

y = (Q_p_/Q_f_) × 100 (4)

The rejection coefficient presented in Equation (5) compares the solute concentration in the inlet flow C_f_ with the solute concentration in the permeate flow C_p_.

R_o_ = (C_f_ − C_p_)/C_f_ (5)

The Spiegler–Kedem model indicates that the transport of solutes through a membrane can be described with the principles of Irreversible Thermodynamics (IT), which relates the fluxes of the solvent and solute with the transport coefficients which, in turn, are independent of solute concentration [57]. For a system made up of two components, water and solute, the TI proposes Equation (6):

J_vo_ = L_p_(Δp − σ × Δπ) (6)

where

J_vo_ = analytical flux,
L_p_ = solvent permeability coefficient (permeability of water in the membrane),
Δp = transmembrane pressure or system operating pressure,
σ = reflection coefficient, and
Δπ = osmotic pressure differential.

The temperature variation during the experimental process generates a variation in J_v_, so it must be adjusted with the Arrhenius model [58] shown in Equation (7).

J_v_ = J_vo_ × e^(−ΔH/R×(1/T − 1/To))^ (7)

where

J_v_ = analytical flux with temperature correction.

#### 2.5.2. Statistical Evaluation of the RO Process

To analyze the results obtained, the response surface graph was constructed. This graph allowed observing the best combination of operating variables, finding the best scenario for the Zn concentration by RO operated at low pressures. Identifying the optimal working ranges implies energy savings during operation without reducing separation efficiency or negatively affecting the RO process. The best operating scenario allows reaching the maximum concentration of Zn and thus recovering it in a second stage after RO.

### 2.6. Evaluation of Zn Concentration by RO

This part aimed to estimate the Zn concentration that could be expected from the RO’s experiments in the context of the study area. First, based on the concentrate flow (Q_c_) and the permeate flow (Q_p_), the mean concentration factor (F_C_) of the concentrate in each treatment was calculated from the data of each experiment, as shown in Equation (8).

F_C_ = (Q_c_ + Q_p_)/Q_c_ (8)

Then, the Zn concentration in the concentrate (C_c_) was determined based on the global rejection rate in each treatment and the annual mean Zn concentration (C_0_) in the water of the sampling point with the highest concentration of Zn as the feed concentration. The C_c_ will be calculated with Equation (9).

C_c_ = C_0_ × F_C_ × (R_o_/100) (9)

Finally, relating the feed flow (Q_f_) in L/h of the RO process and the Zn concentration in the concentrate (C_c_), the estimated annual Zn concentration quantity (Zn) was calculated with a unit transformation factor in kg/year. The previous is established in Equation (10).

Zn_r_ = C_c_ × Q_f_ × 8736 (10)

## 3. Results

In this section, the monitoring results carried out in Milluni will first be discussed. Secondly, the results of the experimentation operating with low pressures in the RO scale pilot will be presented. Thirdly, the estimation of Zn recovery will be shown.

### 3.1. Milluni Monitoring

Milluni monitoring was carried out according to the specifications detailed in Section 2.2. The monitoring results (Table 4) in the Milluni area show that in Point 1 there are no Zn values that exceed the Bolivian Standard 512 and the values established by the EPA. However, Point 2 and Point 3 present values that exceed what is stipulated in the previous regulations. The above agrees with the acidic pH values and high conductivities in Points 2 and 3. Point 2 is the one with the highest Zn values. The maximum value found is 69.9 mg Zn/L which would exceed the permitted limits 14 times. In this sense, the values of Point 2 will be taken as a reference point to assess how much Zn can be recovered in Milluni; this will be explained later in Section 3.3. Figure 2 shows the three points monitored in Milluni.

### 3.2. Experimental

**Figure 4:**
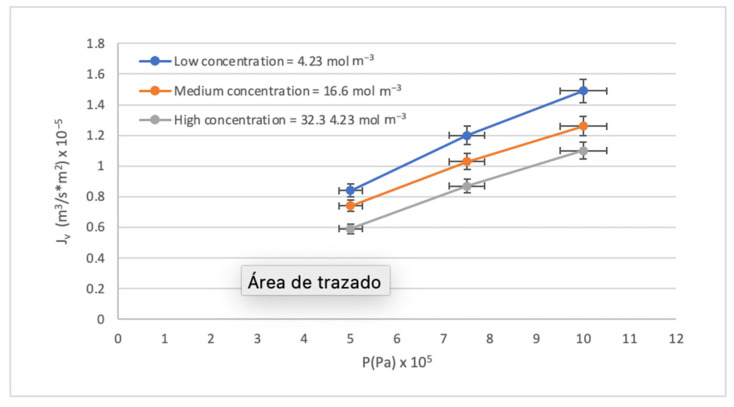
Effect of the P on the J_v._

The results of the pilot-scale RO system experimentation with synthetic waters following the experimental design proposed in Section 2.4. are presented in Table 5. The most notable result can be observed in the Global Rejection Rates obtained since they are all above 98.83% despite having worked with pressures below the range recommended by the supplier. The flux presented adequate behavior; this is described in depth in Figure 4.

To analyze the RO pilot system performance, Section 3.2.1 and Section 3.2.2 present mathematical and statistical evaluations of the results obtained from the experimental part.

#### 3.2.1. Mathematic Evaluation

The behavior of the RO membrane during the separation process can be evaluated as a function of two variables: the analytical flux with temperature correction (J_v_) and the operating pressure (P). In Figure 4, J_v_ and P are directly proportional, like a membrane’s normal behavior. Three concentration levels were used, and an inverse proportionality was identified between the J_v_ values and the concentration of the synthetic waters. The high concentration curve is below the low and medium concentration curves. The above is explained by the phenomenon of concentration by polarization (CP), which occurs on the surface of the membrane and affects mass transfer. It is known that when working at low pressures, the behavior of flux with pressure tends to be linear, so the physical compaction of the membrane is not observed when solute concentrations are low.

**Figure 5:**
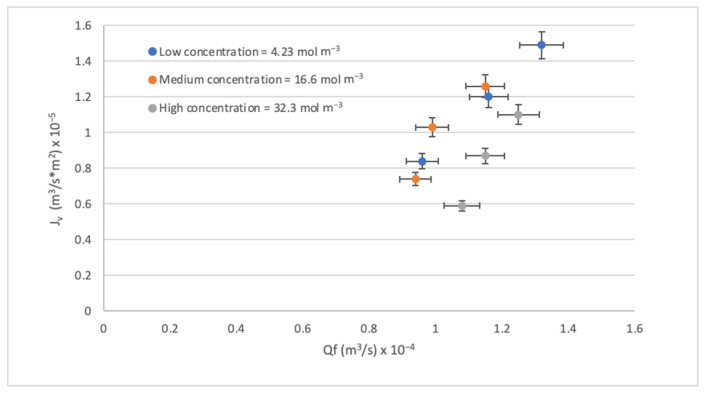
Effect of the Q_f_ on the J_v._

**Figure 6:**
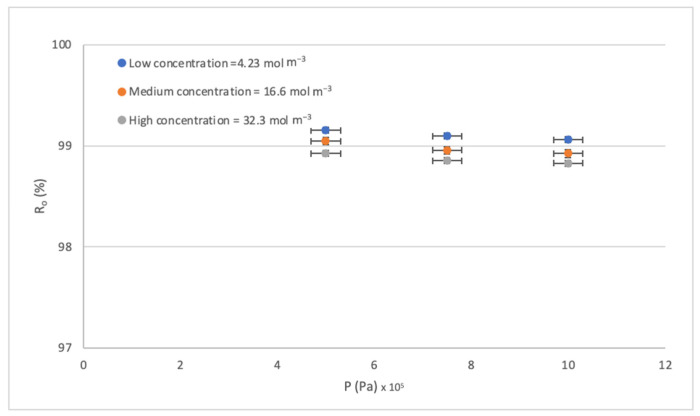
Effect of the P on the R_o._

Another way to physically evaluate an RO process is by observing the effect of feed flow Q_f_ on the J_v_. As seen in Figure 5, increasing the Q_f_ with low concentrations increases the velocity, which increases the mass transfer coefficient by increasing the J_v_. On the contrary, the increase in concentration also causes the CP to increase, generating a decrease in the J_v_. Other studies have also described the above [59,60]. Another important thing to highlight from Figure 6 is that the effect of Q_f_ on the J_v_ is not noticeable in low and medium contractions but is more noticeable in high contractions. A drop in J_v_ can also be observed as the concentration increases, but in any case, the J_v_ maintains the same trend with all concentrations.

**Figure 7:**
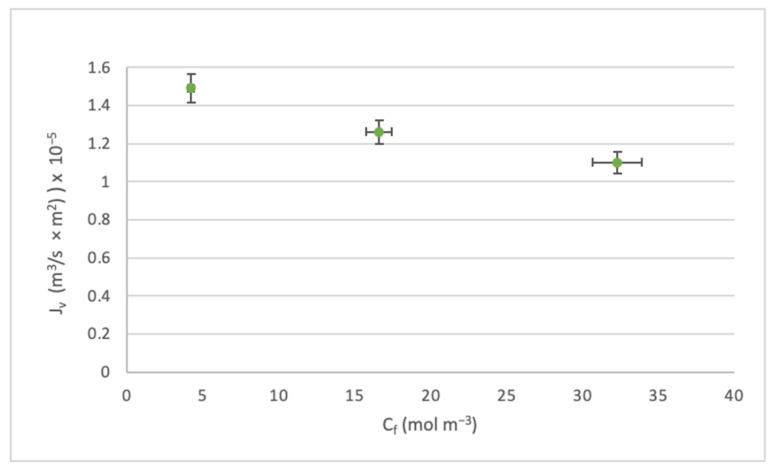
Effect of the C_f_ on the J_v._

The effect of pressure P on the rejection rate R_o_ is perhaps the least studied because high rates are normally achieved working in the pressure range established by the supplier. So, leaving the pre-established range is a way to seek sustainability by reducing the energy cost that RO entails. Figure 7 shows that the rejections obtained are not those expected by the increase in operating pressure. Therefore, obtaining high efficiencies without operating in high-pressure conditions opens up. This innovation within the operation can complement other studies on achieving energy efficiency within membrane filtration processes [61,62]. By increasing the concentration gradient, what is observed is that more of the solute passes through the diffusion phenomenon, which implies lower rejection rates. The above can be seen in Figure 7; the R_o_ at a lower concentration is higher. It is also observed that Ro remains slightly constant within the error levels. On the other hand, when the pressure increases, a double phenomenon is observed in the transport mechanism. First, as the pressure increases, the pores of the membrane close, and fewer ions pass through. But, simultaneously, as more water passes, it drags the solutes out of the solution, causing a slight decrease in the Ro. The above is seen in Figure 6, but it is a very insignificant effect.

**Figure 8:**
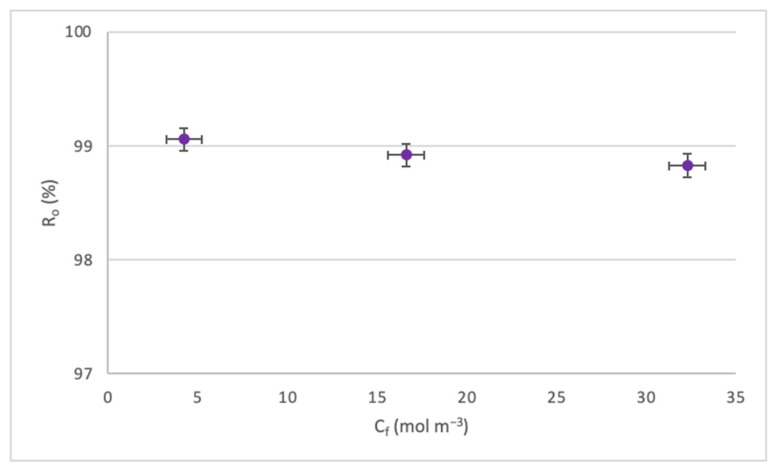
Effect of the C_f_ on the R_o._

In the same line as the study [57], the analysis was carried out on the effect of the feed concentration C_f_ on the flux J_v_ and the rejection index Ro. Figure 7 and Figure 8 were constructed based on the scenarios where the highest pressure level was operated. However, both the medium and low pressures had the same trends.

Through Figure 7, it can be seen that when C_f_ increases, J_v_ decreases. This is due to a decrease in mass transfer and concentration polarization near the membrane boundary layer.

Figure 8 shows a slight decrease in Ro for the increase in C_f_. This occurs since, as the concentration increases, the CP process approaches the boundary layer of the membrane, producing a decrease in solute rejection. It must be highlighted that all rejections remained above 98%.

#### 3.2.2. Statistical Assessment of Response Variables

**Figure 9:**
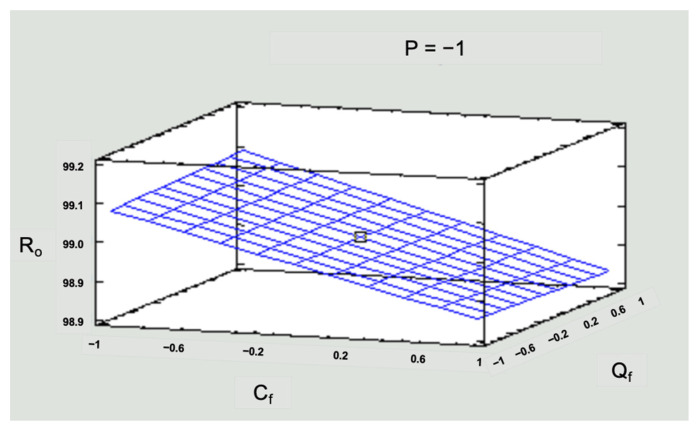
Response surface graph for R in the low-pressure operating scenario.

**Figure 10:**
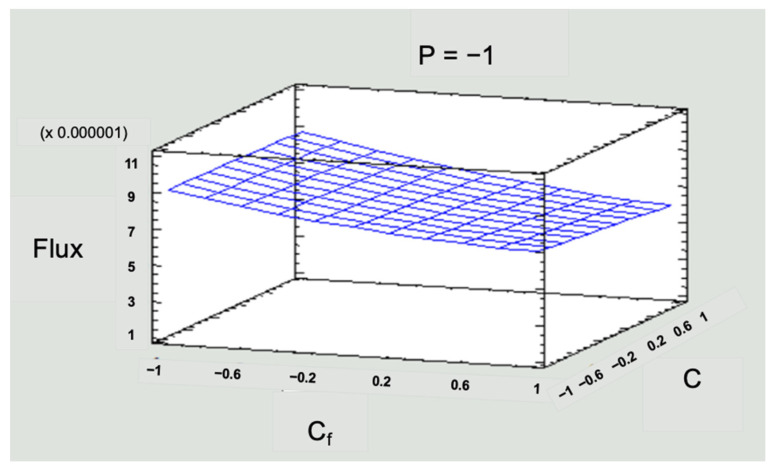
Response surface graph for flux in the low-pressure operating scenario.

A statistical evaluation of the process allows for optimizing the operating conditions of the RO to find the most sustainable scenario in terms of energy costs to concentrate Zn. This study worked in critical pressure conditions; the pressures were below the range established by the supplier. Figure 9 and Figure 10 present the response surface graphs, where the interaction of the three input variables (concentration, pressure, and feed flow) is observed for each response variable (R_o_ and J_v_). Response surface graphs were constructed for all scenarios, but Figure 9 and Figure 10 represent the scenarios with low-level pressures as an example of critical scenarios.

To simplify the numerical information in Figure 9 and Figure 10, values of −1 for the low level, 0 for the medium level, and 1 for the high level were used for the three operating variables in the system: pressure (P), solution concentration (C_f_), and inlet flow (Q_f_).

Figure 9 shows that the highest rejection rate was achieved for low concentrations, 99.15%. However, the Ro obtained for the medium and high concentrations corresponds to 99.05% and 98.92%, respectively. This is significant for the study area since concentrations that exceed national and international standards were simulated. So, a response greater than 98% would be beneficial to solve the simulated pollution problem. Regarding flow, working with a low, medium, or high flow does not cause a significant direct effect on the response variable R_o_.

In Figure 10, the flux values were kept below that recommended by the manufacturer of the membrane module, which implies a vital advantage such as energy savings when working with low pressures and would not decrease the separation efficiency. It could also prolong the life of the equipment since it will not be operating at full capacity. Regarding operation, it could be assumed that there will be fewer fouling problems in the membranes, implying less frequent chemical cleaning. The above indicates the possibility of reducing operating costs in an RO process if working with flux values lower than those stipulated by the supplier.

### 3.3. Estimation of Zn Concentration

Regarding the results of Zn measurements at the three sampling points, the Milluni Chico Lagoon presented the highest Zn concentration with a mean annual concentration of 48.67 mg/L (Table 4). Thus, to evaluate the sustainability of Zn concentration, the lagoon is the better-adapted site due to its historic mining pollution [46]. To estimate the annual Zn concentration (Table 6), a constant annual Zn concentration was assumed in Milluni, and the metal concentration by the RO process was defined by the global rejection rate.

As shown in Table 6, the quantities of Zn that could be recovered from the Milluni Chico Lagoon vary from 195.52 to 266.21 kg Zn/year. It should be considered that this estimated concentration corresponds to using only one membrane module in a laboratory-scale RO pilot plant. Scenario 3 (low solution concentration C_f_, high-pressure P, and medium-income flow Q_f_) allows the highest amount of Zn concentration. Nevertheless, these conditions are the most energy consuming of all the scenarios, and with the lowest amounts of Zn in the area that can be concentrated in an RO module. The results of this study show that, operatively, the RO conditions in scenario 7 (high solution concentration C_f_, low-pressure P, and high-income flow Q_f_) are the most sustainable but reduce the quantity of Zn concentration by 27% compared to scenario 3.

It should be considered that despite working with pressures below what the supplier establishes in the nine scenarios proposed (Table 5), the most sustainable scenario for the Zn concentration can still be found in scenario 7. The above may not be representative of energy savings in a laboratory-scale application, but it can greatly impact large-scale sustainability. The concentration of Zn using a laboratory-scale RO pilot operated at low pressures is possible. It may be promising on a large scale due to the energy savings in operation. However, metal recovery must be carried out in the second stage after RO by already-known methods such as chemical precipitation, adsorption, electrochemical treatment, coagulation–flocculation, and photocatalytic separation [63]. The method to choose for Zn recovery must be evaluated according to the particularities of each specific context.

## 4. Discussion

The contamination of water sources is a global problem that must be addressed to avoid a water shortage for consumption. Conventional WWTPs treat wastewater, but they also harm the air and soil due to the emissions and waste generated during treatment, in addition to requiring energy and certain inputs for their operation, as established [64]. For WWTPs to be sustainable, treatment processes must be optimized and outputs recovered to give them added value if it is possible.

According to [65], a mathematical and statistical analysis of a process provides us with information to optimize the technology and maximize responses. The mathematical evaluation of the RO process helped to observe if, when operating with low pressures below that recommended by the supplier, the behavior of the membrane is adequate and if the separation efficiency is maintained in congruence with [66]. The statistical evaluation of the RO process exposed the optimal operating ranges to maximize efficiency and, at the same time, Zn concentration. During the concentration of Zn by RO, the solubility product of the metal or metals in the mixture must be taken into account to establish a maximum concentration in the system and thus avoid an early fouling of the RO modules.

Regarding the chemical speciation of Zn within the Milluni Chico Lagoon, the measured pH, around 3 (Table 5), indicates that the main Zn chemical species in the water is Zn^+2^ [67]. Thus, the chemical form of the metal in the concentrate of the RO process will be the same. The influent of the RO process used in this study was synthetic water (Table 3). In the study area, the presence of other metals in the feed water could affect the Zn recovery during the RO process [68]. To recover Zn from the RO concentrate, different techniques are available, like chemical precipitation, adsorption, electrochemical treatment, coagulation–flocculation, and photocatalytic separation [63]. Chemical precipitation and adsorption are currently the most cost-effective and well-studied techniques to extract metals from the water [69].

In the Bolivian context, economic and technical limits are very important to ensure the sustainability of the Zn recovery process. Thus, even though the methodology is not eco-friendly, chemical precipitation is the better-adapted method due to the high possibility of automatization to industrialize the process [69]. Moreover, taking into account the diversity of metals found in the area (As, Cd, Cr, Fe, Hg, Zn) [46,47], contrary to adsorption techniques, the chemical process allows selective extraction of metals, up to 99.7% efficiency for Zn recovery with di(2-ethylhexyl) phosphoric acid (DEHPA), for example [70,71]. Finally, the cationic free form of Zn in the concentrate facilitates the chemical precipitation of the metal, which confirms the selection of chemical precipitation as a Zn recovery technique associated with the RO process. Taking into account the Zn recovery percentages with chemical precipitation, the estimated quantities of Zn recovery with the pilot would be around 195 kg/year. Relating to the Zn prices presented in Figure 3, this recovery represents a value between USD 500 and 600. Knowing that Bolivia is a country where mining is a central activity of the economy due to its wealth in minerals, the scaling of the pilot on a larger scale and the possibility of taking advantage of other metals would allow better sustainability of the process.

The world is currently in the transition from WWTPs to W&RRFs as established in [20], and to achieve it, research must be deepened into the different water treatment processes. This research studied how to operate the RO sustainably and the feasibility of recovering the pollutants that the OI concentrates in one of its outlets. W&RRFs will be an “ecologically sustainable” technological system and an important component in smart cities.

## 5. Conclusions

This research concludes that it is possible to remove Zn by RO operated at low pressures. The above is corroborated by the metal removal percentages that have been achieved, equal to or greater than 98.83%. The mathematical evaluation showed adequate behavior of the membrane since no operational problems affected the concentration of the metal. Through the statistical evaluation, the most sustainable operation scenario found for Zn concentration from the water through the RO pilot system was scenario 7. The energy savings that can be achieved by operating with low pressures on a laboratory scale are not very important, but on a large scale, it can mean significant savings in operating costs.

RO technology can recover metals from complex mixtures, so the capacity of Zn concentration has to be studied using contaminated water from the Milluni area to evaluate the influence of the metal mixture’s presence on Zn concentration working with the optimized conditions found in this study. As a second stage to recover the metal, considering the context of Bolivia and the treatments available in the literature, the application of chemical precipitation is recommended as a Zn recovery technique associated with the RO process.

This study aims to shift the current paradigm surrounding the economic and environmental impact of advanced water treatment processes by modifying the traditional operation of RO to make it more sustainable and reduce its energy consumption. Additionally, the research examined the most effective method of recovering Zn in Bolivia to promote a circular economy within water treatment plants where this process could be implemented.

## Figures and Tables

**Table 1:** Summary of the sampling protocol designed for Milluni.

Component	Details
Objective	Determine concentration of Zn in the Milluni area.
Monitoring area	The upper part of Milluni, including its lagoons: Pata Khota, Jankho Khota, Milluni Chico, and Milluni Grande.
Sampling points	Three sampling points were determined to monitor the concentration of Zn in Milluni. Point 1: Pata Khota lagoon was defined to identify reference conditions at the beginning of the lagoon system (E 592549; N 8197635). Point 2: Laguna Mulluni Chico, located in the middle of the lagoon system, was defined to determine any signs of deterioration of water quality due to anthropogenic activity (E 589887; N 8193022). Point 3: Laguna Milluni Grande was defined to show if its water has the desired quality for being a dam that supplies two cities in Bolivia (E 589147; N 8190424).
Frequency of monitoring	Three samplings were defined in the dry season and three in the rainy season with intervals of 1 month between samplings.
Parameters to measure	The *in situ* parameters that must be monitored are pH, dissolved oxygen, conductivity, turbidity, and temperature. In addition, concentrations of Zn.
Equipment	HQ40D—Hach Handheld Multimeter for pH, dissolved oxygen, and conductivity. Hach 2100Q Handheld Turbidimeter for turbidity. Agilent Technologies, Madrid, Spain, Model 7700x Inductively Coupled Plasma Mass Spectrometer (ICP-MS) for Zn concentration.
Sampling	For proper sampling and transportation, ISO 5667-1 and ISO 5667-4 were followed [55,56]. The above standards stipulate the necessary sample volumes and the number of repetitions for measuring parameters *in situ*.

**Table 2:** Details of the components used in the pilot-scale RO system.

Item	Model	Brand	Operational Conditions	From
RO membrane module	ULP-2540	Keensen^®^	maximum pressure 4.14 × 10^6^ Pa; 2–10 pH	Changsha, China
Case of the membrane module	2540.300.1	Wave Cyber Co., Ltd.	maximum pressure 4 × 10^6^ Pa	Shanghai, China
Multistage centrifugal/electric pump	2ACM150H	LEO^®^	2 HP; maximum pressure 5.00 × 106 Pa	Zhejiang, China
Flow sensors (FS)	FT-110	Gems Sensors & Controls™	0.5 to 5 L/min 1 to 25 L/min	Plainville, CT, USA
Manometer	AN213.53	WIKA	0–4.5 × 10^6^ Pa	Germany
Arduino	MEGA 2560 R3	ELEGOO	-	Silicon Valley, China
Needle valve	AISI-316	GENEBRE	-	Barcelona, Spain

**Table 3:** Synthetic water concentrations for experiments.

Solution Concentration	Zn (mol m^−3^)	NaCl (mol m^−3^)	Solution Concentration C_f_ (mol m^−3^)
Low level	2.9 × 10^−2^	4.20	4.23
Medium level	8.8 × 10^−2^	16.5	16.6
High level	3.0 × 10^−1^	32.0	32.3

**Table 4:** *In situ* and *ex situ* parameters determined in Milluni.

Sapling Month	Points	Parameters Measured *In Situ*				Parameter Measured *Ex Situ*
		Turbidity	pH	Dissolved Oxygen	Conductivity	Zn
		NTU		mg/L	µS/cm	mg/L
January	Point 1	4.14	6.85	7.31	48.3	0.003
	Point 2	29.3	3.32	5.54	745.0	25.68
	Point 3	11.7	3.03	5.21	1119.0	25.88
March	Point 1	3.21	7.01	7.56	37.8	0.01
	Point 2	20.6	3.54	6.01	800.0	42.08
	Point 3	11.7	3.45	5.59	1080.0	24.26
May	Point 1	1.83	6.70	7.90	38.7	0.033
	Point 2	3.41	2.83	6.59	1723.0	54.85
	Point 3	2.75	2.76	7.06	1246.0	25.71
August	Point 1	3.25	7.37	7.66	64.9	0.005
	Point 2	7.83	2.78	6.41	1966.0	69.91
	Point 3	8.07	2.68	6.68	1442.0	34.06
October	Point 1	3.51	4.60	5.60	71.5	0.01
	Point 2	5.30	2.81	4.99	1718.0	58.06
	Point 3	23.2	2.67	5.67	1486.0	37.21
December	Point 1	2.54	6.50	5.80	65.2	0.02
	Point 2	4.50	3.23	4.45	850.0	41.47
	Point 3	9.80	2.57	4.98	1200.0	32.62

**Table 5:** Input and output variables in the pilot-scale RO experiment.

Nº	Input Variables			Output Variables	
	Solution Concentration C_f_ (mol m^−3^)	Pressure P (Pa)	Income Flow Q_f_ (m^3^/s)	Flux J_v_ (m^3^/s × m^2^)	Global Rejection Rate R_o_ (%)
1	Low	Low	Low	8.37 × 10^−6^	99.15
2	Low	Medium	High	1.20 × 10^−5^	99.10
3	Low	High	Medium	1.49 × 10^−5^	99.06
4	Medium	Low	Medium	7.44 × 10^−6^	99.05
5	Medium	Medium	Low	1.04 × 10^−5^	98.96
6	Medium	High	High	1.26 × 10^−5^	98.92
7	High	Low	High	5.91 × 10^−6^	98.92
8	High	Medium	Medium	8.65 × 10^−6^	98.85
9	High	High	Low	1.08 × 10^−5^	98.83

**Table 6:** Estimated Zn concentration by RO process.

No.	Mean Concentration Factor F_C_	Global Rejection Rate Ro (%)	Annual Mean Zn Concentration in Milluni Chico Lagoon C_0_ (mg/L)	Zn Concentration in the Concentrate C_C_ (mg/L)	Mean Flow Q_f_ of RO Process (L/h)	Estimated Annual Zn Concentration Quantity Zn_r_ (kg/year)
1	1.36	99.15	48.67	65.69	345.89	198.51
2	1.42	99.10	48.67	68.40	420.33	251.17
3	1.56	99.06	48.67	75.06	405.99	266.21
4	1.28	99.05	48.67	61.78	373.45	201.56
5	1.35	98.96	48.67	65.10	357.16	203.12
6	1.41	98.92	48.67	68.02	415.32	246.79
7	1.20	98.92	48.67	57.52	389.11	195.52
8	1.31	98.85	48.67	62.80	383.55	210.43
9	1.44	98.83	48.67	69.02	356.49	214.96

## Data Availability

The original contributions presented in the study are included in the article, further inquiries can be directed to the corresponding author.
